# *QuickStats:* Percentage* of Adults Aged 20–64 Years Whose Blood Cholesterol Was Checked by a Health Professional in the Past 12 Months,^†^ by Race/Ethnicity^§^ — National Health Interview Survey,^¶^ United States, 2011 and 2016

**DOI:** 10.15585/mmwr.mm6645a8

**Published:** 2017-11-17

**Figure:**
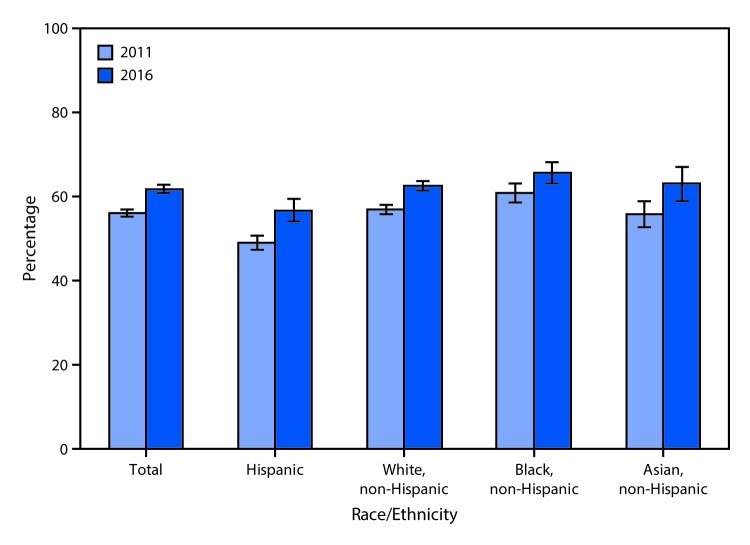
The percentage of adults aged 20–64 years who had a blood cholesterol check by a health professional in the past 12 months increased from 56.0% in 2011 to 61.7% in 2016. From 2011 to 2016, there was an increase in the percentage of adults with a blood cholesterol check among Hispanic (49.0% to 56.7%), non-Hispanic white (56.8% to 62.5%), non-Hispanic black (60.8% to 65.6%), and non-Hispanic Asian (55.8% to 63.0%) persons. In both years, non-Hispanic black adults were more likely than non-Hispanic white adults to have had a blood cholesterol check, and Hispanic adults were the least likely to have had a blood cholesterol check.

**Source:** National Health Interview Survey, 2011 and 2016 data. https://www.cdc.gov/nchs/nhis.htm.

